# Supplementary material for: Common Genetic Variants of The Cardiac Sodium Channel Alter Patient Response to Class 1b Antiarrhythmics
Source: bioRxiv. 2026 Jan 14:2026.01.14.699482. Preprint. [Version 1] doi: 10.64898/2026.01.14.699482 (PMC12871244; doi:10.64898/2026.01.14.699482)
Supplement: 1 [file NIHPP2026.01.14.699482V1-supplement-1.pdf]

bioRxiv preprint doi: https://doi.org/10.64898/2026.01.14.699482; this version posted January 14, 2026. The copyright holder for this preprint (which was not certified by peer review) is the author/funder, who has granted bioRxiv a license to display the preprint in perpetuity. It is made available under aCC-BY 4.0 International license.

Supplementary Data

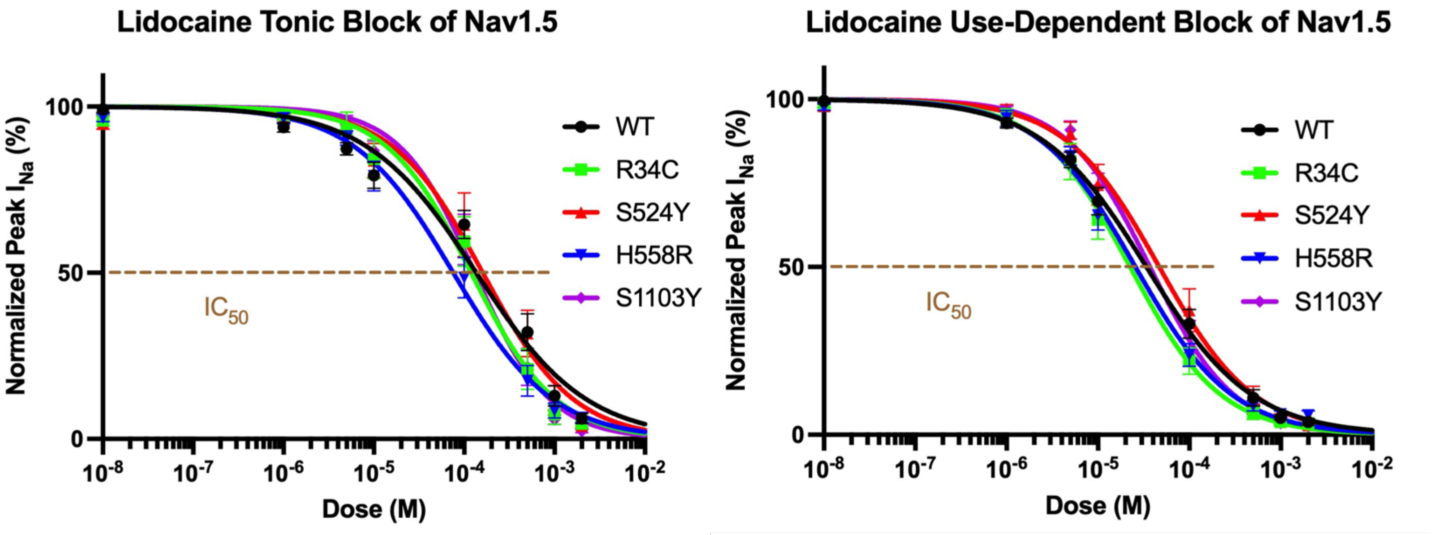

SI Fig. #1: Lidocaine block of Nav1.5 currents in transiently transfected HEK293 cells. A) Lidocaine tonic block measured at 0Hz stimulation. The H558R IC_{50} (75.6±16.6 μM) is significantly left-shifted compared to WT (IC_{50}: 136.1±33.9 μM), p=0.003. The other variants do not exhibit a significant effect (p>0.05). B) Lidocaine use-dependent block measured at 10Hz frequency. The H558R IC_{50} (25.7±4.2 μM) is significantly left-shifted compared to WT (IC_{50}: 33.9±5.5 μM), p=0.03. The other variants do not exhibit a significant effect (p>0.05). Sample size is 8-12 cells per variant. Nav1.5 currents are recorded with automated planar patch clamp.

bioRxiv preprint doi: https://doi.org/10.64898/2026.01.14.699482; this version posted January 14, 2026. The copyright holder for this preprint (which was not certified by peer review) is the author/funder, who has granted bioRxiv a license to display the preprint in perpetuity. It is made available under aCC-BY 4.0 International license.

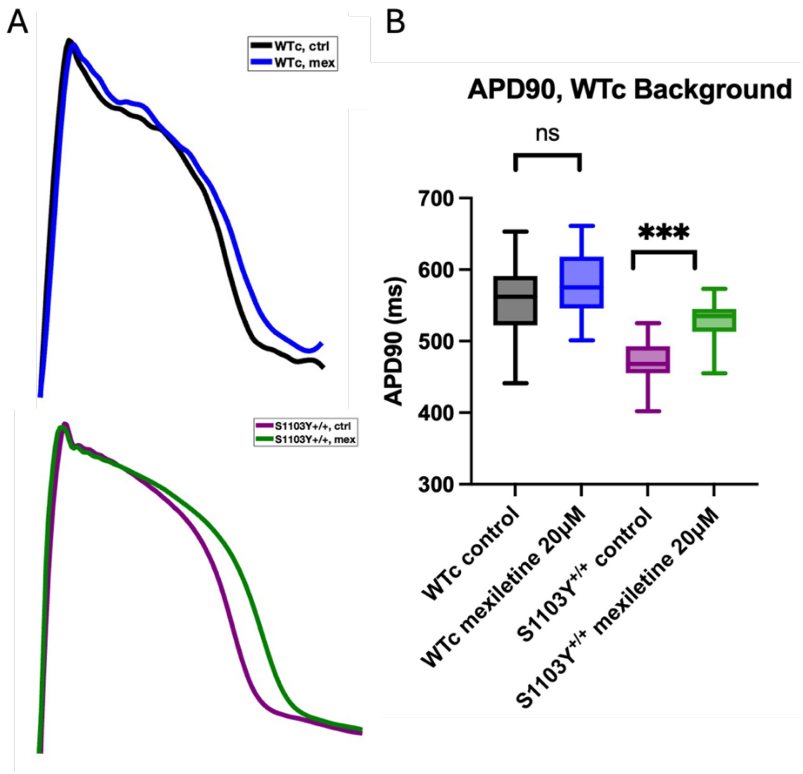

SI Fig. #2 Action potential duration (APD) in WTc background iPSC-CM lines. A) Representative APD_{90} traces measured at 1Hz in control and mexiletine-treated cells. 20μM mexiletine was applied 15 minutes before recording. The Savitzky-Golay filter was applied to all representative traces. B) Mexiletine significantly prolongs the APD90 (+58.9ms) in the S1103Y homozygous line ($p < 0.001$) compared to control. The parent WTc line does not exhibit significant changes in APD_{90} after mexiletine treatment ($p = 0.1$).

bioRxiv preprint doi: https://doi.org/10.64898/2026.01.14.699482; this version posted January 14, 2026. The copyright holder for this preprint (which was not certified by peer review) is the author/funder, who has granted bioRxiv a license to display the preprint in perpetuity. It is made available under aCC-BY 4.0 International license.

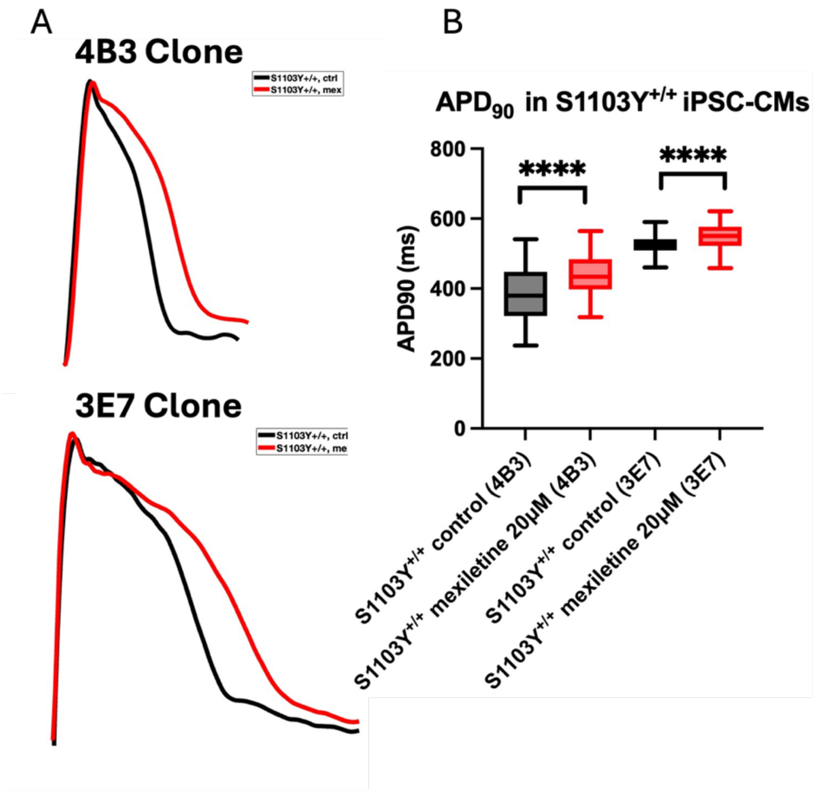

SI Fig. #3: Action potential duration (APD) in two clones of S1103Y$^{+/+}$, MVET-55 background iPSC-CMs. A) Representative APD_{90} traces measured at 1Hz in control and mexiletine-treated cells. 20µM mexiletine was applied 15 minutes before recording. The Savitzky-Golay filter was applied to all representative traces. B) Mexiletine significantly prolongs the APD_{90} by +48.6ms in the 4B3 clone ($p < 0.001$) and by +47.7ms ($p = 0.008$) in the 3E7 clone compared to control.
